# Physical simulation of the influence of the original rock strength on the compaction characteristics of caving rock in longwall goaf

**DOI:** 10.1098/rsos.220558

**Published:** 2022-09-14

**Authors:** Yan Qin, Nengxiong Xu, Yuxi Guo, Jinyang Li, Wenbin Han

**Affiliations:** ^1^ School of Engineering and Technology, China University of Geosciences (Beijing), Xueyuan Road 29, Beijing 100083, People's Republic of China; ^2^ Shanxi Traffic Layout Reconnaissance Design Institute Co. Ltd, Wuluo Street 27, Taiyuan 030032, People's Republic of China

**Keywords:** original rock strength, caving rock, compaction characteristics

## Abstract

The compaction characteristics of broken rock in a caving zone have a significant impact on the movement law of overburden and the prediction of surface subsidence. The mechanical properties of the broken rock were clearly affected by the original rock strength of the roof. Based on the similarity theory, the ‘quartz sand-gypsum-lime-water’ mixed material was used to make similar samples of original rocks with different strengths, and the compaction mechanical behaviour of broken loose rock masses with different original rock strengths was studied. The results show that (i) the greater the original rock strength of broken rock, the shorter the initial compaction stage, the earlier the transition and stable compaction stages and the lower the degree of compaction; (ii) the initial deformation modulus and ultimate axial strain had a linear relationship with the original strength of the broken rock; and (iii) under different axial pressures, the deformation modulus increased with the increasing original rock strength of the broken rock. The tangent modulus and axial stress change approximately linearly, the secant modulus and stress change linearly, and the tangent modulus and secant modulus exhibit an exponential/hyperbolic relationship with the strain. The research results have high engineering application value for using numerical method to predict the mechanical behaviour of roof rock mass with different strength in coal mining and analysing the surface subsidence.

## 1. Introduction

In the process of coal mining, roof rock within a certain height breaks into gravel of different sizes and accumulates into new bulk rock materials. The rock stratum in this area is also known as the caving zone. With increasing mining length, the overlying rock gradually collapses and the broken rock in the caving zone becomes compacted. The compaction mechanical properties of caving rock have a significant impact on the movement law of the overburden (such as the step distance of strata breaking, the height of fracture development and the range of surface subsidence) and the permeability characteristics of the goaf [1,2]. Therefore, studying the mechanical properties of broken rock is of great significance for rock pressure prevention, water inflow evaluation and surface subsidence prediction.

Under the action of an overlying load, the gap between the broken rocks in the caving zone was small, and the contact increased. Macroscopically, the volume is compressed and the mechanical properties are gradually strengthened [3,4]. Previous studies have shown that the compaction process of broken rocks can be divided into three stages: initial compaction, gradual compaction and stable compaction [5]. However, the reduction in the block size and rearrangement of fillings are the main factors affecting the compaction process [6].

The experimental study showed that the stress–strain curve of broken rock in the caving zone is exponential [7], and the secant modulus is linear with stress [8]. The greater the original rock strength, the smaller the final deformation, and the strain value in the initial compaction stage was not affected [9]. The different strengths of the roof rock can be divided into three grades: weak, medium hard and hard. According to the empirical formula and relevant test results, the compaction stress–strain curves of broken rock masses formed by different roof strengths also differ [10].

As a mechanical parameter that can be obtained directly from engineering sites, roof strength is widely used in the calculation of roof collapse steps and prediction of fracture zone height. Previous studies on the mechanical properties of broken rocks have mostly used real rock for crushing, and indoor tests have been conducted. Owing to the different mineral contents of the roof rock in different mining areas, there are clear differences in strength. However, to explain the influence of strength on the mechanical properties of broken rocks, a direct comparison is difficult. In this study, the 'quartz sand-gypsum-lime-water' mixed material was used to make similar samples of original rocks with different strengths by controlling the material ratio based on the similarity theory. Taking strength only as the test variable, the influence of different original rock strengths on the compaction characteristics of the broken rock was studied.

## 2. Methods

### 2.1. Material properties of the physical model

A traditional mixture of quartz sand, gypsum, lime and water was selected as the model material. Quartz sand is the skeleton, and gypsum and lime are cementitious materials. In the cementitious materials, the mass ratios of gypsum and lime were fixed at 7 : 3. The mass of water added to the materials was 10% of the total mass of the quartz sand, lime and gypsum (see table 1 for a detailed proportion scheme).

**Table 1.** Mass proportion of model materials.

serial number	quartz sand : gypsum : lime : water
Case 1	100 : 14 : 6 : 12
Case 2	100 : 21 : 9 : 13
Case 3	100 : 28 : 12 : 14
Case 4	100 : 35 : 15 : 15
Case 5	100 : 42 : 18 : 16
Case 6	100 : 49 : 21 : 17

**Figure 1.**
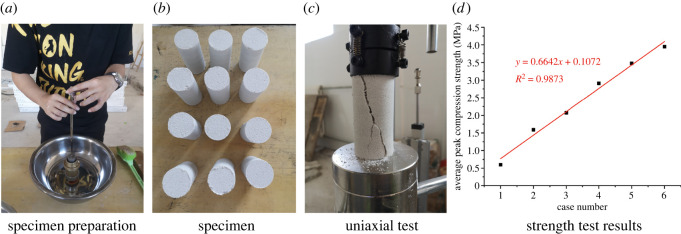
Making cylindrical samples of model materials with different ratios and testing the strength.

According to the above ratio, a cylindrical sample with a diameter of 39.1 mm and height of 80 mm was made with a geotechnical test mould (figure 1*a*), and three samples were set for each ratio (figure 1*b*). After the sample was prepared, it was dried in indoor ventilation for 15 h, then the quality and size were measured, and a uniaxial compressive test was carried out (figure 1*c*; see table 2 for detailed test results), and the relationship between the peak compressive strength and the ratio is plotted in figure 1*d*. The peak strength of the sample increased linearly with increasing proportion of cementitious materials.

**Table 2.** Original test results of the density and strength of different materials.

serial number	specimen number	mass (g)	average mass (g)	density (kg/m^3^)	peak load (kN)	average peak load (kN)	average peak strength (MPa)
1	1-1	169	168.7	1.76 × 10^3^	0.53	0.72	0.60
	1-2	168			0.69		
	1-3	169			0.93		
2	2-1	174	172.3	1.79 × 10^3^	2	1.91	1.59
	2-2	172			2.06		
	2-3	171			1.67		
3	3-1	182	178.3	1.86 × 10^3^	2.3	2.49	2.07
	3-2	177			2.98		
	3-3	176			2.18		
4	4-1	179	180.0	1.87 × 10^3^	2.64	3.49	2.91
	4-2	180			4.47		
	4-3	181			3.37		
5	5-1	179	181.3	1.89 × 10^3^	3.97	4.17	3.48
	5-2	184			4.54		
	5-3	181			4.01		
6	6-1	183	184.0	1.92 × 10^3^	3.58	4.74	3.95
	6-2	185			5.57		
	6-3	184			5.07		

### 2.2. Particle size and gradation of broken rock in caving zone

According to the analysis of the particle size of broken rock in the caving zone in [11], the maximum particle size of broken rock can reach more than 1.2 m, but the mass proportion is relatively small, and most particle sizes are distributed from 0.4 to 1.2 m (table 3). In this test, 0.3, 0.6, 0.9 and 1.2 m are selected as the characteristic particle sizes of broken rock in the caving zone, and the geometric similarity constant is set as 30. The particle sizes of the broken rock in the model test were 1, 2, 3 and 4 cm. In addition, to eliminate the boundary effect, the maximum particle size of the broken rock mass should be less than one-fifth of the inner diameter of the test cylinder [12]. Therefore, the diameter of the test cylinder was designed to be 20 cm, and its height was 40 cm.

**Table 3.** Test results of the distribution proportion of crushed stones with different particle sizes in the goaf [11].

particle size (m)	0–0.05	0.05–0.1	0.1–0.4	0.4–0.6	0.6–0.8	0.8–1.2	>1.2
mass proportion (%)	3.164	4.465	7.482	35.554	16.257	16.264	15.814

**Figure 2.**
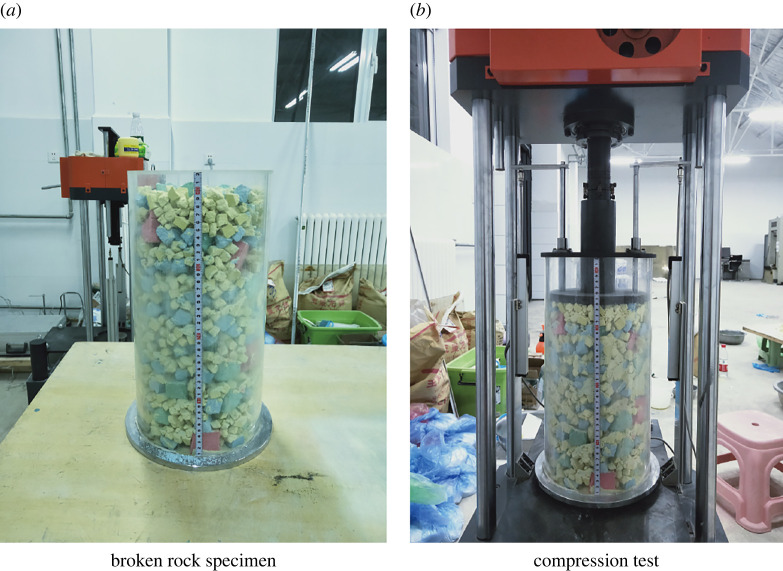
Preparation and compression testing of broken rock samples with different grades.

According to the fractal theory of fractured rock masses [13], the value range of the fractal dimension *D* of fractured rock masses in caving zones is generally 2.0–2.7. The ratio of the mass of rock particles with a particle size of less than *D* in the fractured rock mass to the total mass of the samples can be expressed as follows:

2.1 $$\frac{M_{d}}{M_{t}}={\left(\frac{d}{d_{M}}\right)}^{3-D},$$

where *D* is the particle size of the broken rock mass; *M*_d_ is the mass of particles with a particle size less than *D*; *M*_t_ is the total mass of the sample; and *d*_M_ is the maximum particle size of particles. In this test, *D* was set to 2.4, which unifies the rock grading characteristics to facilitate the study of the influence of strength on the compaction mechanical behaviour. Broken rock samples were prepared according to the above particle size and grading methods, and compaction tests were performed, as shown in figure 2. The displacement loading speed used in this test was 0.5 mm s^−1^.

## 3. Results and discussion

### 3.1. Stress–strain relationship and compactness

**Figure 3.**
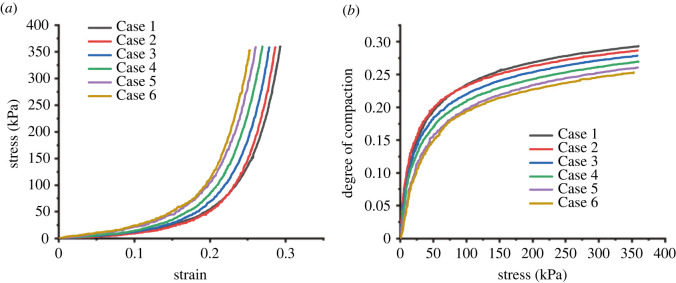
Compaction stress–strain curve (*a*) and compaction degree change curve (*b*) of broken rocks with different strengths.

According to the test data, the stress–strain curves of broken rocks with different ratio strengths under the same grading conditions were drawn, as shown in figure 3*a*, and the curve of compactness versus stress is shown in figure 3*b*. The stress–strain curves of the broken rocks show an exponential/hyperbolic growth relationship. In the initial compaction stage, the original rock strength had little effect on the stress–strain curve of the broken rock. When the strain reached approximately 0.07, the stress–strain curves were different, specifically: (i) the greater the original strength of the broken rock, the faster the curve rises. Under the same strain condition of broken rock, the greater the original rock strength, the greater is the axial load required. (ii) When the axial strain reached approximately 0.18, the broken rock of Case 6 experienced a deformation transition period, and the deformation transition period was completed when the axial strains of Case 1 and Case 2 reached approximately 0.22. (iii) With increasing stress, the broken rock gradually became compacted. The greater the original rock strength, the smaller the axial limit strain and the lower the degree of compaction.

The previous study [14] regarded a broken rock mass in the caving zone as a granular material. Based on the theory of rock mass mechanics, the variation relationship between the porosity and dilatancy coefficient of broken rock mass in the caving zone with deformation is deduced, and the stress–strain equation of deformation in the caving zone is proposed (formula (3.1)), which is also called the Salamon model.

**Figure 4.**
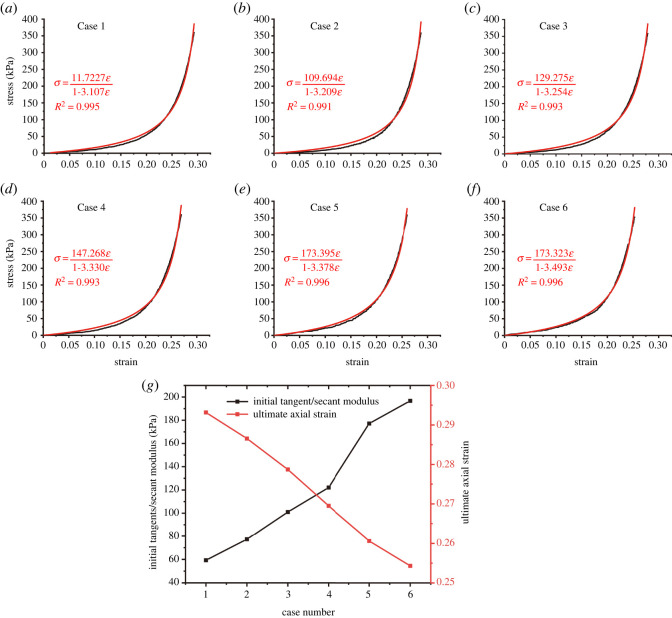
(*a*)–(*f*) Fitting the stress–strain curve of broken rock with different strengths by the Salamon model and (*g*) the relationship between the initial deformation modulus, axial limit strain and strength.

3.1 $$\sigma =\frac{E_{0}\varepsilon }{1-\varepsilon /\varepsilon_{m}},$$

where *E*_0_ is the initial tangent modulus at the initial stage of loading, *ε*_m_ is the ultimate axial strain of compaction, *σ* is the axial stress of the specimen, and *ε* is the axial strain of the sample. The Salamon model was used to fit the stress–strain curves of the broken rock samples with different strength ratios, as shown in figure 4*a–f*. The Salamon model fits the stress–strain curves of broken rocks with different strengths well. The relationship between the initial deformation modulus and the ultimate axial strain in the fitting parameters and the ratio is shown in figure 4*g*. With an increasing proportion of cement in the material (from Case 1 to Case 7), the strength of similar materials increased, the ultimate axial strain of the broken rock decreased linearly and the initial deformation modulus increased gradually, which was also approximately linear.

### 3.2. Deformation characteristics

**Figure 5.**
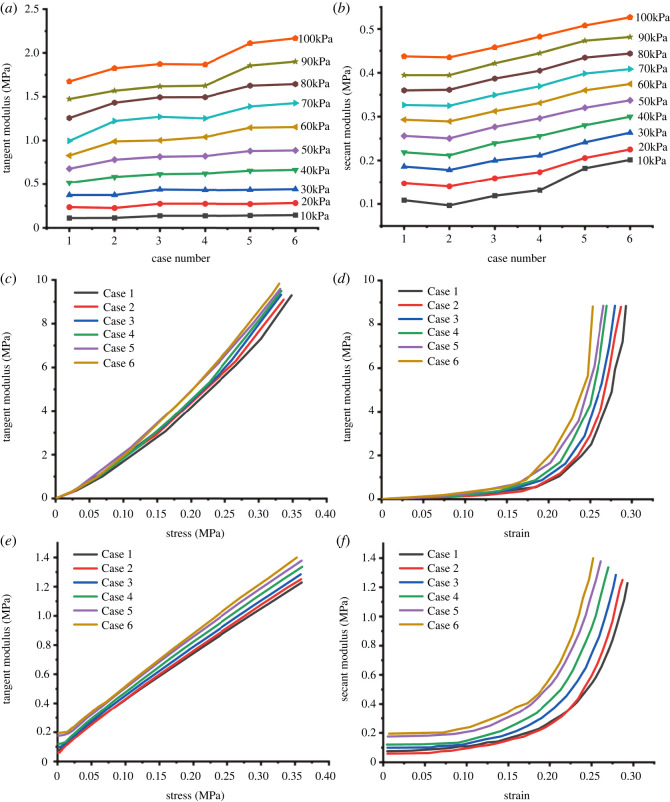
(*a*) Relationship between the tangent modulus and different ratios of model material under different axial loads, (*b*) relationship between the secant modulus and different ratios of model material under different axial loads, (*c*) relationship between the tangent modulus and stress of broken rock with different ratios of model material, (*d*) relationship between the tangent modulus and strain of broken rock with different ratios of model material, (*e*) relationship between the secant modulus and stress of broken rock with different ratios of model material and (*f*) relationship between the secant modulus and strain of broken rock with different ratios of model material.

Figure 5 shows the relationship between the tangent modulus, secant modulus, stress and strain during the loading of broken rocks with different ratios.

When the axial stress is small (10–30 kPa), the tangent moduli of the broken rock with different strengths are equal. Figure 5*a*. With increasing axial stress (greater than 40 kPa), the tangent modulus of the broken rock with a high original rock strength increased faster. Under different axial stress states, the higher the original rock strength of the broken rock, the greater the secant modulus (figure 5*b*). The increase in the secant modulus of the broken rock with different original rock strengths with increasing axial stress was basically the same.

As shown in figure 5*c–f*, under the same axial stress or axial strain, the greater the original rock strength of the broken rock, the greater the tangent and secant moduli. The tangent modulus was approximately linear with the stress, but the change relationship exhibited an evident upward trend (figure 5*c*). The secant modulus has an obvious linear relationship with stress (figure 5*e*). Both the tangent modulus and secant modulus change exponentially with the strain or can also be called a hyperbolic change relationship (figure 5*d,f*).

## 4. Conclusion

In this study, the model test method was used to prepare broken rock samples with different strengths according to a certain gradation. The influence of the mechanical strength on the compaction characteristics of broken rocks was studied. The main conclusions are as follows:

(1) The stress–strain curves of broken rocks with different original rock strengths have three stages: initial compaction, transition compaction and stable compaction. The greater the original rock strength, the shorter the initial compaction stage of the broken rock, the earlier the transition and stable compaction stages, and the lower the degree of compaction.
(2) The initial deformation modulus and ultimate axial strain have a linear relationship with the original strength of the broken rock. The stress–strain curves of broken rocks with different strengths can be well fitted by the Salamon model.
(3) Under different axial pressure conditions, the deformation modulus increased with increasing original rock strength of the broken rock. During the entire compaction process, the tangent modulus and axial stress changed approximately linearly; however, the curve exhibited an upward trend. The secant modulus changes linearly with the stress. The tangent modulus and secant modulus have an exponential/hyperbolic relationship with the strain.

## Data Availability

The research data supporting this publication are available from the Dryad Digital Repository at https://doi.org/10.5061/dryad.02v6wwq5p [15].
